# Addressing the Grand Challenge of atmospheric carbon dioxide: geologic sequestration vs. biological recycling

**DOI:** 10.1186/1754-1611-5-14

**Published:** 2011-11-02

**Authors:** Ben J Stuart

**Affiliations:** 1 Ohio University, Athens, Ohio 45701, USA

**Keywords:** carbon dioxide, capture, sequestration, storage, biological recycling, biomass, algae, sustainability

## Abstract

On February 15, 2008, the National Academy of Engineering unveiled their list of 14 Grand Challenges for Engineering. Building off of tremendous advancements in the past century, these challenges were selected for their role in assuring a sustainable existence for the rapidly increasing global community. It is no accident that the first five Challenges on the list involve the development of sustainable energy sources and management of environmental resources. While the focus of this review is to address the single Grand Challenge of "develop carbon sequestration methods", is will soon be clear that several other Challenges are intrinsically tied to it through the principles of sustainability. How does the realm of biological engineering play a role in addressing these Grand Challenges?

## The Problem: Atmospheric Carbon Dioxide

Human activities are impacting the natural world at a global scale leading to accelerating non-linear system response. Among the Greatest Engineering Achievements of the 20^th ^Century, electrification captured the number one spot, followed by two transportation methods; the automobile and the airplane [1]. While these technologies clearly have irreversibly changed the way we conduct our lives on a daily basis in a positive sense, it has been made possible only through the combustion of cheap, carbon-based fuels.

Unfortunately, extraction and utilization of fossil fuels have changed the environmental landscape. From the air we breathe, to the water we drink and the land we rely upon for all of our material resources, changes (some might say irreversible) to our global ecosystems have been documented that suggest future generations cannot continue "life as we know it". Entire libraries could be filled with publications that have been dedicated to documenting the physical, chemical and biological impacts that by-product emissions have had on global climates and ecosystems. As a call to action in response to these and other critical issues facing the global community, the National Academy of Engineering unveiled their list of Grand Challenges for Engineering, including the management of carbon in our atmosphere [2].

**Figure 1:**
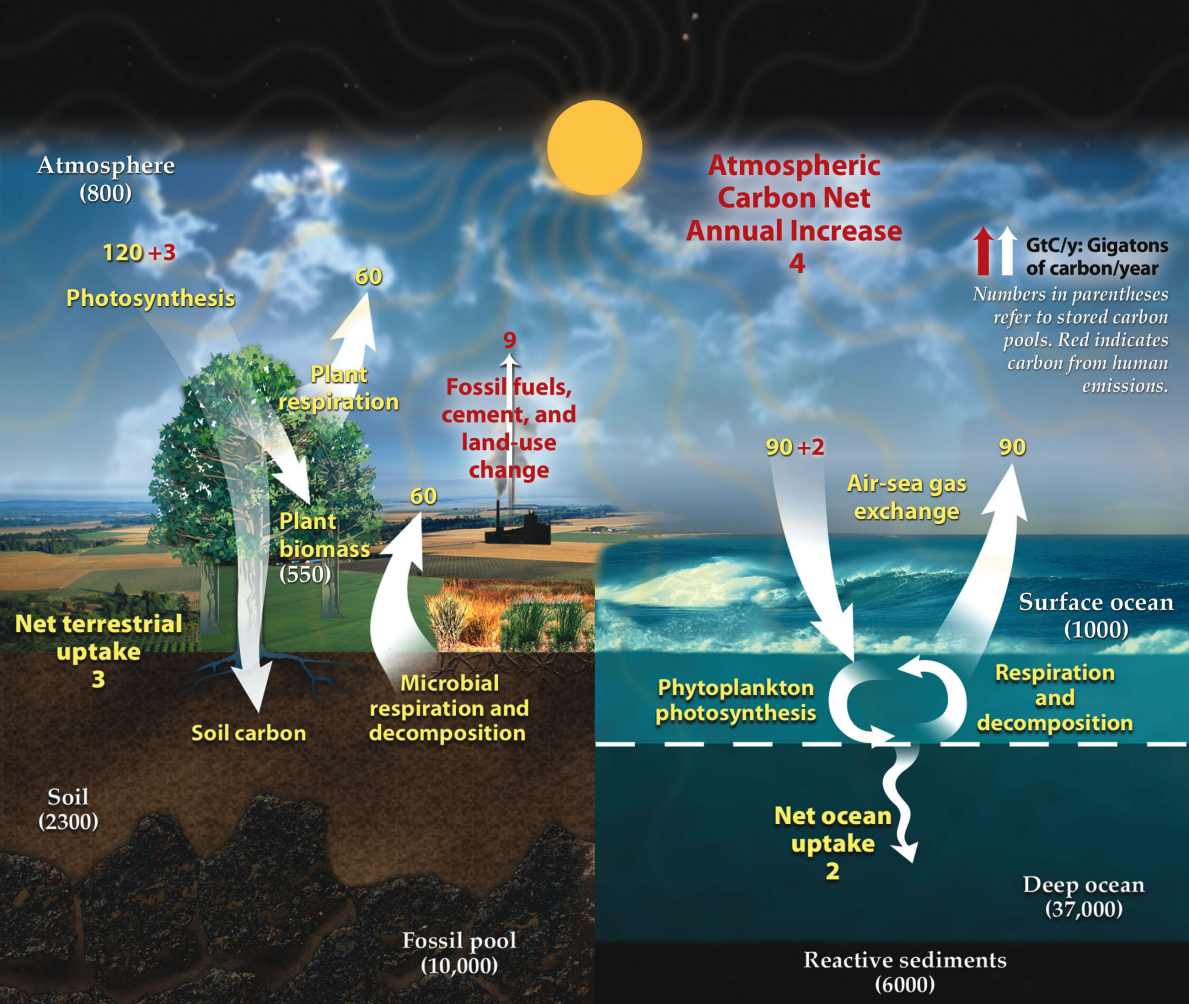
**Carbon storage and annual carbon fluxes in natural and engineered environments **https://public.ornl.gov/site/gallery/originals/BioComponents_Carbon.jpg. Image courtesy of U.S. Department of Energy Genomic Science program http://genomicscience.energy.gov.

A few comprehensive reviews have cited hundreds of scientific reports for those interested in understanding historic and current trends and impacts [3-5]. Carbon dioxide (CO_2_) measurements in the atmosphere have increased from 280 ppm in pre-industrial years to almost 390 ppm today [6], and the vast majority of stored CO_2 _is currently sequestered in the deep oceans [7]. Figure 1 is a simplified representation of the global carbon cycle where the numbers in parentheses are estimates of the primary carbon reservoirs in gigatons carbon (Gton C = billion metric tons of carbon). Natural fluxes are shown in the diagram as yellow numbers, while human contributions are identified through red text. It is interesting to note that, with over 57,000 Gton C sequestrated in natural reservoirs, the estimated 9 Gton C emitted annually due to human activities results in a net increase of 4 Gton C into the atmosphere each year.

**Figure 2:**
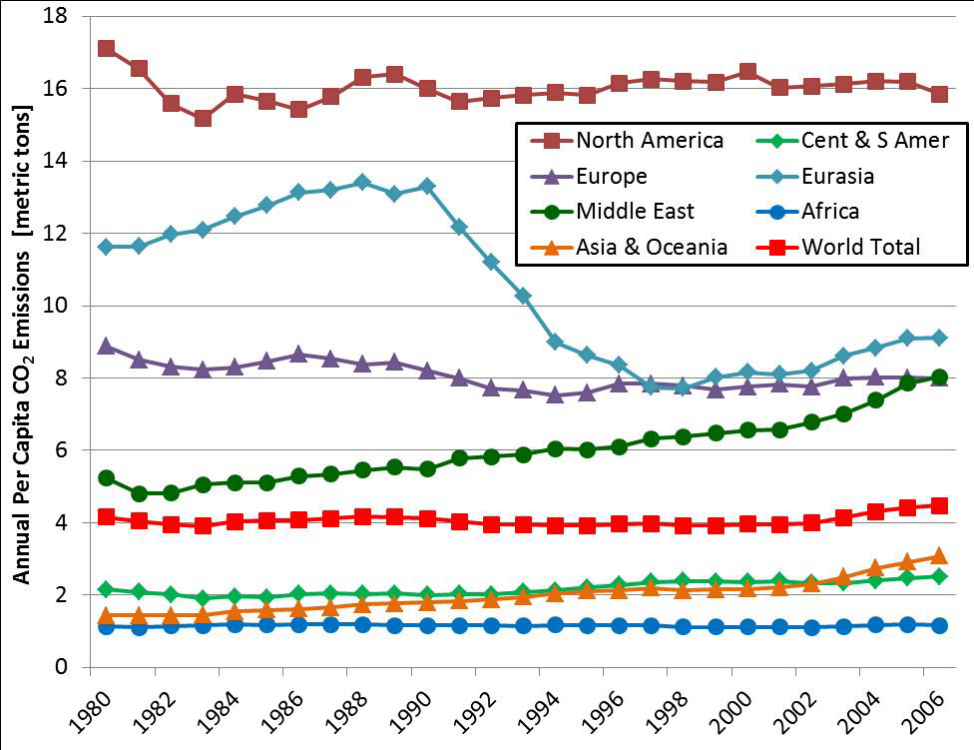
**Annual per capita CO_2 _emissions (metric tons) by geographic region, 1980-2006 [data extracted from **http://www.eia.gov/iea/carbon.html]. Note that "Eurasia" is defined as the U.S.S.R. through 1991, and Russia plus the former Soviet states from 1992-2006. The shift in per capita emissions stems from the development of the former Soviet states into independent, sovereign nations.

The World Bank estimates that global per capita CO_2 _emissions have held relatively steady since 1980 at 4-4.5 metric tons per year (Figure 2). However, accounting for increasing world populations (currently estimated at 6.9 B), this results in 31 Gton CO_2 _emitted to the atmosphere per year [8]. Since CO_2 _is 27.3% carbon, that is equivalent to 8.5 Gton C emitted per year. Notably, the U.S. per capita emission rate is nearly five times the global rate, averaging 20 metric tons per person per year during the same time frame. When population is accounted for, currently the U.S. (≈ 309 M people) is responsible for 20% of the global CO_2 _emissions, second only to China (≈ 1.34 B people) at 22.3% and ahead of the entire European Union (≈ 501 M people) at 14% [8]. Although a vocal minority of the population disagree as to the ultimate impact, the overwhelming majority of the scientific community points towards increasingly volatile and extreme local variations in weather patterns and a general increase in the global average temperature [3,4,9].

The CO_2 _molecule is introduced to the atmosphere at specific geographic locations around the globe, and this occurs disproportionately near densely populated areas of developed countries due to power generation and transportation emission sources. However, while measureable variations exist in local, national, and regional concentrations, the worldwide transport of CO_2 _results in impacts to global ecosystems. Therefore, when assessing options for mitigation, sequestration or remediation efforts for CO_2_, the location of facilities must be addressed and may be required to be at or near significant emission points.

## Geologic Sequestration

Although geologic sequestration of CO_2 _has been rigorously researched, the complexity and number of interactions demands continued study. Comprehensive reviews of critical issues are beyond the scope of this paper, but are ubiquitous in the literature [10-15]. The primary attractiveness of geologic sequestration of CO_2 _is that current infrastructure for power generation utilizing fossil fuels can continue to operate while sustainable alternative technologies are developed. In this way, existing emissions will be maintained, or even reduced, even as new demands are imposed by population growth and as additional countries continue to develop. When the useful life of the existing power infrastructure expires, the hope is that all new systems will be zero-to-low carbon emitters, or at least be fitted with carbon capture and sequestration (CCS) technology.

In order to make a significant impact on atmospheric concentrations, many advocating CCS offer an initial target of 1 Gton of carbon per year. Since CO_2 _is 27.3% carbon, that would require the sequestration of 3.7 Gton of CO_2_, or roughly 12% of the currently emitted mass. Presently, CO_2 _is injected into crude oil reservoirs to enhance oil recovery (EOR) through increased reservoir pressure and reduced fluid viscosity. While roughly one quarter of the injected gas is trapped in the reservoir, the remaining gas is recycled and returned to the reservoir during subsequent injections to increase overall sequestration efficiency. Unfortunately, only 48 Mton CO_2 _per year are currently being used in this way, and only 25% of that amount is from anthropogenic sources [16].

**Figure 3:**
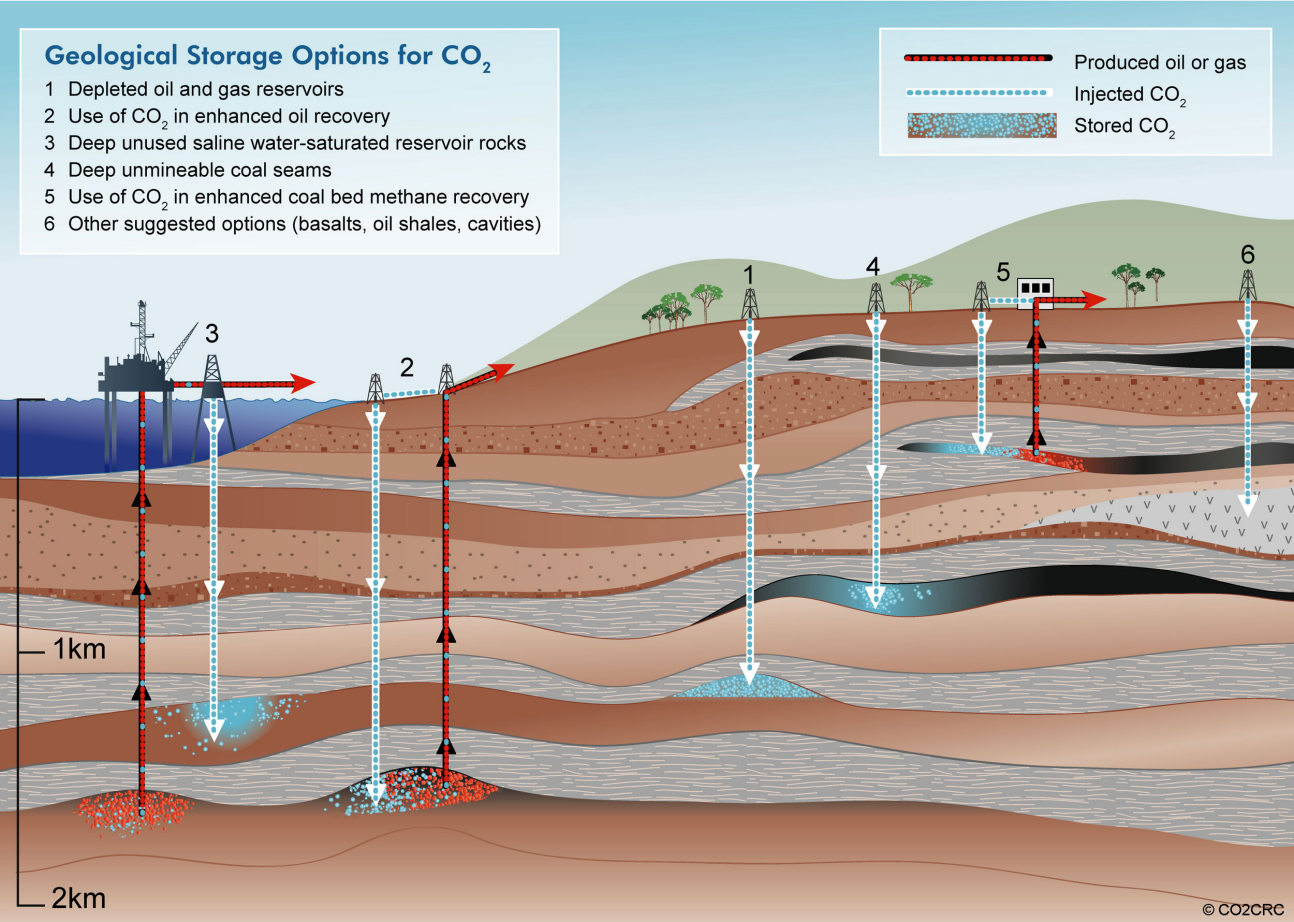
**Geologic storage options for carbon dioxide as recovered during CCS activity **http://www.co2crc.com.au/images/imagelibrary/stor_diag/storageoptions.jpg. Image copyright and provided courtesy of CO2CRC.

A 2005 IPCC report had estimated that worldwide potential for CCS could be as high as 2000 Gton CO_2 _[10], however the 2010 NETL Carbon Sequestration Atlas III reported a low estimate for saline formation capacity in North America alone at 1653 Gton CO_2_, with a high estimate of 20,213 Gton [17]. Figure 3 identifies additional repositories, such as oil and gas reservoirs, coal-bed methane (CBM) formations, basalt formations, organic-rich shale basins, and ocean sediment deposits [17]. These storehouses would need to be identified, evaluated and verified for integrity prior to large-scale, long-term use.

In order to evaluate the reality of geological CCS of anthropogenic CO_2_, let's consider the current emissions from coal fired power plants in the U.S. At 20% of the global 31 Gton CO_2_/yr, the U.S. generates about 6.2 Gton CO_2_/yr. Coal fired power plants are a readily identifiable point source and account for about 30% of this total, which is roughly 2 Gton CO_2_/yr. If we assume the U.S. goal was to sequester 50% of the emitted CO_2 _from these sources, this would result in the CCS of 1 Gton CO_2_, which is equivalent to 10^12 ^kg/yr. Since reservoir densities average about 500 kg/m^3^, this would require delivering 2 × 10^9 ^m^3^/yr at reservoir density to the subsurface. By comparison, the U.S. consumes about 21 Mbbl of crude oil per day, which is equivalent to 3.22 × 10^11 ^gal/yr, or about 1.2 × 10^9 ^m^3^/yr. In essence, the U.S. would have to process twice the volume of our current crude oil infrastructure just to address half of our current CO_2 _emissions from coal fired power plants. Remember, this is an amount which is equal to just 16% of the total CO_2 _emissions in the U.S. Ultimately, one has to acknowledge the fact that geologic CO_2 _sequestration truly represents a Grand Challenge.

Another issue that is often raised is a question as to the integrity of the selected reservoirs on a geologic basin scale. Often the question of CO_2 _leakage is related to the potential for pre-existing faults and/or wells, and transport out of secure reservoirs via regional groundwater flows [18]. On the side of stable sequestration are trapping mechanisms that convert the compressed CO_2 _into mineral fractions that are structurally sound and permanently retained in the subsurface. The most important of these mechanisms is capillary trapping, which can be described as the trapping of a non-wetting phase in a porous medium as discontinuous pore-scale droplets by capillary forces. This phenomenon has been studied extensively due to its relevance for oil recovery and contaminant remediation [19,20]. In these applications, the motivation is the extraction of the trapped phase (oil and/or contaminant). In the context of geological carbon storage, the objective is to maximize trapping, and thus sequestration of the target species (CO_2_).

**Figure 4:**
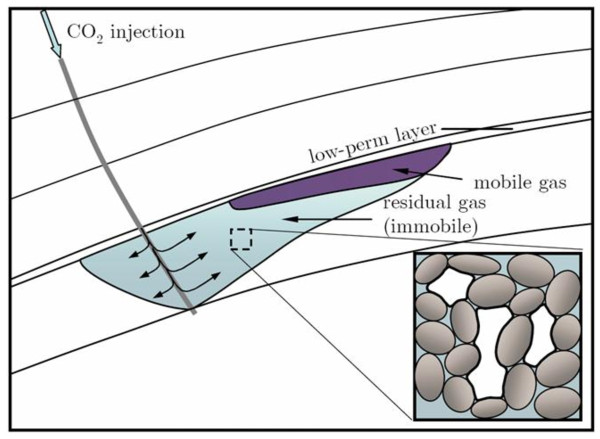
**Schematic of plume behavior and residual material retention due to capillary trapping during injection of CO_2 _in deep aquifers **http://juanesgroup.mit.edu/news[ 21].

In this process, CO_2 _is injected into a pre-assessed geological formation, where it forms a continuous plume. As the injected CO_2 _migrates towards the surface due to fluid buoyancy, ambient groundwater flows into the void space previously occupied by the CO_2 _in a process known as reimbibition. When this occurs, a fraction of the migrating CO_2 _will be rendered immobile within the pores of the geologic strata by capillary forces (Figure 4), and remains stable (minimal leakage to the atmosphere) so long as the strata force balance remains unchanged. This process can be enhanced through injection of additional brine [21,22].

The reservoir capacity estimates mentioned previously can be derived from fluid dynamics, which may also delineate the extent of plume migration, as well as the plume footprint of the injected CO_2 _at the ultimate position. An example on the basin-level could be realized at the Powder River basin where extensive characterization offers an opportunity to select the optimal formation into which the CO_2 _may be injected. After identification of system boundaries, if there is a scenario with the potential of CO_2 _reaching the surface, then that formation is removed from further consideration. Additional boundary conditions may apply, such as pre-determined flow-paths that must be avoided. Once all constraints on boundary conditions are established, an array of anticipated well locations can be identified and the ultimate CO_2 _footprints mapped.

To ensure permanence of CO_2 _sequestration at the basin-scale, coupling the flow profiles with geo-mechanics is needed to assure the absence of fault activation, and continuous assessment of the fate of the CO_2 _in the 4-D seismic surface deformation would be required. Finally, all this must be accomplished within the boundaries of an effective regulatory framework to guide and react to the findings of the theoretical and applied research in geological sequestration of CO_2_. Again, while rigorous, theoretical science is aggressively being pursued, and field case studies are providing valuable data, high-volume, long-term storage in geologic layers is many years, possibly decades away. Further, given the global number and distribution of both stationary and mobile sources, the reality of CCS of a significant fraction of the anthropogenic carbon dioxide is suspect. Finally, CCS does not address CO_2 _currently in the atmosphere, and any portion of ongoing sources not captured will only serve to increase atmospheric concentrations.

## Biological Recycling

Nature already has an extremely efficient method for managing carbon dioxide [7]. Photosynthesis has been credited by many with the transformation of a carbon-based atmosphere to an oxygen-based one, while in the process generating much of the biomass necessary to contribute to the vast quantities of fossil fuels we rely so heavily upon today. The photosynthetic process requires light, water, carbon dioxide, nutrients (typically nitrogen and/or phosphorous are limiting) and a biological agent to convert the CO_2 _into energy, biomass and oxygen. It is interesting to note that managing solar energy, water resources, carbon dioxide, and the nitrogen cycle are among the 14 Grand Challenges identified by the National Academy of Engineering. Further, two of the top three Challenges (energy from solar and fusion sources) address the need for development of economically viable sustainable energy resources [2].

**Figure 5:**
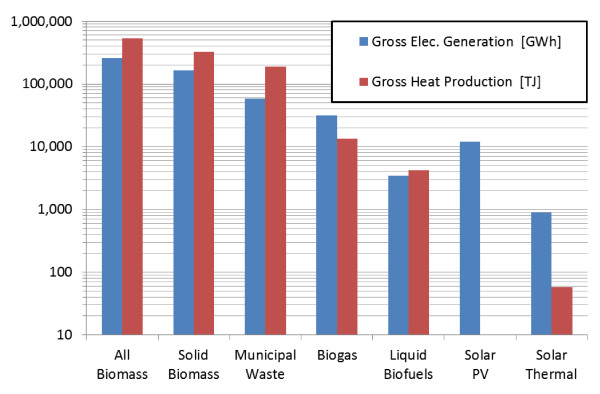
**Global gross electricity generation and heat production from various biomass and solar sources, 2008 [data extracted from **http://www.iea.org/stats/renewdata.asp?COUNTRY_CODE=29].

While photovoltaic cells or solar thermal systems are usually first to come to mind when contemplating the energy that could sustainably be recovered from the sun, it is important to remember that all photosynthetic organisms use the sun as a primary energy source as well. Figure 5 compares global energy generation (electricity and heat) via several biomass and solar sources in 2008. Clearly, there is an array of biomass choices, most of which currently provide significantly more energy than from other solar-based energy technologies. However, it should be noted that several recent advances in solar PV and solar thermal technologies have significantly reduced the cost of these technologies, greatly increasing their potential to supply future global energy demands.

Autotrophic biological agents convert solar energy into chemical energy through the conversion of carbon dioxide, and subsequently store this energy in a relatively stable organic form. In reality, fossil fuels derived from the deposition of organic matter throughout time differ from modern biofuels only in the amount of time that has elapsed from the time the solar energy was captured and stored. The generation and use of biofuels is not carbon neutral [23], however they do shift the carbon balance from one of continuously re-introducing previously stored carbon to one where current carbon in the atmosphere can be recycled into an energy source an indefinite number of times. As renewable energy sources are advanced and utilized to a greater extent in the production of biomass feedstocks, the carbon balance will continuously shift to approach carbon neutrality.

Additional considerations for the light requirements of photosynthetic agents include the number of light-days per year, light intensity, and light cycle. In the U.S., the 300+ sun days per year and high levels of solar insolation in the desert and coastal southwest, respectively, makes that geographic region attractive for any engineered photosynthetic cultivation system [24,25]. Fortunately, not all autotrophic strains suitable for biomass feedstock development need (or even tolerate) full sun [26]. For example, many strains of algae utilize pigmentation to regulate light levels, and some may reject 90+% of all incident photons. Further, cultivation systems that incorporate light management and/or redistribution systems can take advantage of the vast majority of incident light, allocating precise (wavelength differentiated), optimal intensities to all locations in the growth environment. Finally, in excessive light environments, there is a potential for photo-inhibition and UV damage that could have a negative impact on overall mass productivity.

While the southwest region of the U.S. can boast in its availability of sunlight, that area is severely lacking in the water resources so critical to successful, large-scale aquaculture. Fortunately, algae are easily isolated from, or able to be adapted to a wide array of water sources including, but not limited to fresh, brackish, saline, municipal and agricultural wastewaters, certain industrial discharges, and even acid mine drainage. And while they may be cultivated in reclaimed wastewaters, they may also be employed in the reclamation (excess nutrient removal) of those wastewaters [27]. The downside of traditional ponds, lagoons and raceways is that they provide an opportunity for water to evaporate, and if these types of cultivation systems are located in the desert southwest, significant loss of water volume to the atmosphere can be expected. Further, as this water evaporates, any media salts present will concentrate, potentially shifting the growth dynamics of the culture. To combat these losses, covered ponds will be required in certain locations, and while the covers have the potential added benefit of filtering harmful ultraviolet radiation, this additional level of control comes at increased capital and maintenance costs.

The availability of CO_2 _for biological recycling is a non-issue as carbon dioxide is ubiquitous, a non-respecter of source and is relatively equally distributed around the globe. Using the commonly accepted stoichiometric equation for photosynthesis:

$${\text{H}}_{2}\text{O}+\text{C}{\text{O}}_{2}+\to \text{C}{\text{H}}_{2}\text{O}+{\text{O}}_{2}$$

one mole of CO_2 _yields one mole of carbohydrate (CH_2_O), or equivalently 1.47 kg of CO_2 _is consumed for every 1 kg of carbohydrate produced. However, if we use a stoichiometric equation substituting the average result from the ultimate analysis of aquatic biomass for the carbohydrate term [28], we get:

$$1.11\;{\text{H}}_{2}\text{O}+1.58\;\text{C}{\text{O}}_{2}+\to {\text{C}}_{1.58}{\text{H}}_{2.22}\text{O}+1.64\;{\text{O}}_{2}$$

where now 1.87 kg of CO_2 _are consumed for every 1 kg of aquatic biomass produced.

Most biological systems operate quite efficiently at ambient CO_2 _concentrations and mass productivity may be increased for some species when photosynthetic organisms are placed in elevated CO_2 _environments, while other species show declining growth [29,30]. For aquatic species (i.e. algae and micro-algae), most of the decline in growth rate in cultivated systems can be attributed to a decrease in pH due to the formation of carbonic acid during sparging with high CO_2_-containing gas. Further, mass transfer of CO_2 _into an aqueous phase is also dependent on such parameters as gas-phase concentration, interfacial area available for mass transfer, as well as solution properties such as temperature, pH, and the presence and concentration of dissolved salts.

Nutrients required for biological growth typically focus on nitrogen and phosphorus, although many media also contain salts and trace minerals. Fortunately, many of these chemicals can be found in municipal and agricultural wastewaters, and these waste streams are easily accessible worldwide. Additional nutrients could be made available through implementing the concepts of industrial ecology to create "energy campuses" in which waste streams are exchanged and processed into beneficial feedstocks for complementary processes. For example, anaerobic digestion could be employed to convert organic waste streams that are currently being managed through land disposal into a biogas. The gas can be fired to produce electricity, while the nutrient liquor that remains after digestion is nearly an ideal source of fertilizer that could be used to feed terrestrial or aqueous photosynthetic organisms [31]. Further, many autotrophic species also have the potential to fix nitrogen from the atmosphere, thus a well-planned ecosystem would be able to supply critical nutrients over long time frames.

Selecting the appropriate biological agent is primarily dependent on photosynthetic efficiency and the ability for the produced biomass to provide a value product or to serve as a biomass feedstock. As plants provide global populations with food, feed, medicines and nutraceuticals, among many other valuable products, the biological agent and value product can be tailored to meet the needs of any local population. Algae have received much recent attention as a potential biomass feedstock for advanced biofuel production due to its high growth rates, lack of competition as a primary food/feed source, ubiquitous geographic presence allowing for adaptation to a nearly infinite number of growth environments, and the extreme diversity of extractible, high-value co-products [32,33].

It is difficult to estimate the biomass potentially available as a general feedstock without assuming much about market viability in a world of constrained energy reserves. However, recent studies evaluating biofuel potentials estimate almost 550 M dry tons per year (tpy) would be available in the US in the year 2020 from agricultural and woody residues, terrestrial fuel crops grown on currently idle croplands, and animal and municipal solid wastes [34]. This potential has been estimated to increase to 1.366 B tpy when accounting for sustainable recovery from both agricultural land (998 M tpy) and forestland (368 M tpy) by the year 2050 [35]. This amount of biomass has the energy equivalent of 3.8 B barrels of oil, which was approximately 54% of the US consumption in 2010.

Targeted biomass production, such as algae farming for biofuel feedstocks, has recently gained in popularity and is estimated to realistically replace 17% of US oil imports when located in geographic regions with available water resources [36]. That potential increases to 48% of US petroleum imports dedicated to transportation if productivity is maximized, but would require 5.5% of contiguous US land area and would consume nearly 3 times the amount of water currently used for agriculture [36]. However, oil available from lipid extraction represents only a fraction of the total energy potential of algal biomass (additional options will be addressed below).

If the estimated 24 M acres of available crop land were used to produce algae at the realistic target of 30 g/m^2^/day (equivalent to 50 ton/acre/yr), an additional 1.2 B tpy of biomass could be generated with an oil energy equivalent of 3.3 B barrels, or 47% of the US consumption in 2010. At the previously stated ratio of 1.87 kg CO_2 _yielding 1 kg biomass, this amount of biomass would consume 2.24 Gton CO_2 _per year; an amount equivalent to 12% more than the output of all US coal-fired power plants, and 36% of the total US emissions. If advances in cultivation technologies increase productivity to the high estimate of 50 g/m^2^/day identified during the US DOE's Aquatic Species Program [32], the potential increases to 3.73 Gton CO_2 _consumed per year, or 60% of the US total emissions.

So what is the best method for recovering the energy potential from biomass? Clearly, oil producing vegetation (including algae) may be processed for oil extraction which could be made into biodiesel (a fatty acid methyl ester, or FAME) through simple transesterification or upgraded to synthetic diesel, which is chemically equivalent to petroleum diesel. However, energy potentials may take liquid, gas, or solid forms, and an array of processing technologies may be employed to transform biomass into the most appropriate fuel for specific applications. In addition to direct combustion or oil extraction and upgrading, thermal-chemical conversion technologies such as pyrolysis, liquefaction, gasification, Fischer-Tropsch Synthesis (FTS), and catalytic processing are continuously evolving to be able to process the variety of available biomass feedstocks [36,37]. Current biological processing technologies go beyond traditional anaerobic digestion for methane production to include direct production of fuels such as ethanol, butanol and hydrogen through novel fermentation or enzymatic processes [38-40].

## Summary and Engineering Challenges

Addressing atmospheric carbon dioxide truly is a Grand Challenge, however technological approaches are available to address current and future emissions and it would be naïve to assume a single approach would be sufficient to address all concerns. Clearly, our rate of consumption and the efficiency at which we do so are paramount considerations that must first be addressed when developing energy policy going forward. Global development and population growth will only increase the impacts currently seen in our environment, and sustainable practices must be implemented to ameliorate current stressors while we implement technologies to mitigate or even eliminate future emissions. A carefully orchestrated combination of technology development to limit carbon emissions from future energy production, CCS to address current and future emissions of systems still using fossil fuels, and enhanced biological recycling of captured and atmospheric CO_2 _will be necessary to have a significant impact.

This opens wide the door of opportunity to all engineering professions, but perhaps none more so than biological engineers for the development of biomass cultivation and processing technologies. Needs in this field include the identification and development of optimized species, increasing yields through genetic characterization, development of novel, low-cost culturing systems, advancing downstream processes to accommodate multiple feedstocks, and addressing technology availability to the global community. Holistically coupling carbon recycling with the global concerns of water resource allocation, the availability of clean drinking water, managing nutrient cycles for nitrogen and phosphorous that will impact food availability and quality, and sustainable energy production for a world population currently at 7 billion may be the only way to assure a prosperous and sustainable future for all.

## Competing interests

The author declares that he has no competing interests.
